# JAK3 inhibition: what potential for the future?

**DOI:** 10.1186/2047-1440-2-S1-S6

**Published:** 2013-11-20

**Authors:** Christophe Legendre

**Affiliations:** 1 Service de Transplantation Rénale Adultes, Hôpital Necker, Assistance Publique-Hôpitaux de Paris, INSERM U 845, 149 rue de Sèvres, 75015 Paris, France; 2 Université Paris Descartes, Sorbonne Paris Cité, 12, rue de I’Ecole de Médicine, 75006 Paris, France

**Keywords:** kidney transplantation, JAK3, tofacitinib

## Abstract

JAK3 inhibition with the CP-690,550 compound has an immunosuppressive potency in murine models, nonhuman primates and humans. This drug blocks STAT5 activation in most T-cell subpopulations but less effectively in T-regulator cells. In low to moderate risk human kidney transplant recipients, combined with mycophenolate mofetil, steroids and an induction with basiliximab, CP-690,550 proved as effective as calcineurin inhibitors with regard to prevention of acute rejection but better than calcineurin inhibitors with regard to preservation of kidney function and histology. However, at the same time, an increased incidence of overimmunosuppression consequences (cytomegalovirus, BK virus and lymphoproliferation) was observed and led to discontinuation of this specific drug development in kidney transplantation.

## Introduction

The results of kidney transplantation have improved dramatically over the past 20 years – although much progress has been made with regard to short-term results (1-year patient and graft survival), the attrition rate afterwards has changed minimally if at all [1]. In the meantime, immunosuppression became rather homogeneous combining a calcineurin inhibitor (mostly tacrolimus), an inosine-5’-monphosphate dehydrogenase inhibitor, low-dose or no steroids and induction with either basiliximab (low-immunological risk patients) or Thymoglobulin^®^ (high immunological risk patients; Genzyme, Paris, France) [2,3]. The validity of this choice was further consolidated by publication of the Symphony study [4]. However, although the anti-rejection efficacy of this immunosuppression is well known, its numerous side effects (diabetes mellitus, nephrotoxicity, diarrhea, tremor, hypertension, and so forth) are also common! In particular, the responsibility of calcineurin inhibitor-induced nephrotoxicity in chronic allograft dysfunction is currently less obvious while under immunosuppression-induced chronic antibody-mediated rejection is becoming the leading cause of graft loss [5,6]. There is therefore a need for new immunosuppressive drugs that would display at least the same efficacy (and hopefully greater efficacy in high immunological risk patients) but a significantly more acceptable long-term safety profile. In this review we will focus on JAK3 inhibition, a potentially very promising new drug category [7,8].

## JAK3 inhibition

The Janus kinases (JAKs) are a family of four cytoplasmic tyrosine kinases that participate in the signaling of members of the cytokine receptor common gamma-chain family. The JAKs were initially named ‘just another kinase 1 and 2’ (since they were just two of a large number of discoveries in a PCR-based screen of kinases) but were ultimately named ‘Janus kinases’ [9]. The name was taken from the two-faced Roman god of doorways, beginnings, change and transition, Janus, because the JAKs possess two almost identical phosphate-transferring domains. One domain exhibits the kinase activity, while the other negatively regulates the kinase activity of the first.

**Figure 1:**
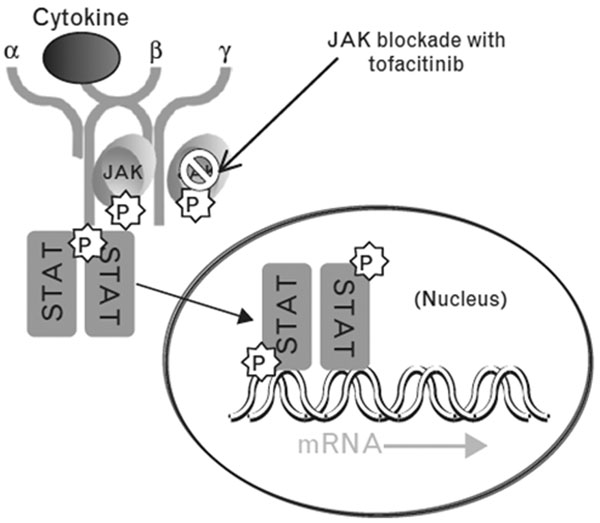
**Cytokine signaling of Janus kinases.** Binding of a cytokine to its receptor activates the associated Janus kinase (JAK), which then phosphorylates (P) the receptor and provides a docking site for signal transducers and activators of transcription (STATs). In turn, STATs become phosphorylated and translocate to the nucleus to regulate gene expression. From [8].

Schematically, there are four JAKs: JAK1, JAK2, JAK3 and tyrosine kinase 2. The activation of JAK occurs by a ligand–receptor interaction that results in signaling through the phosphorylation of cytokine receptors and the creation of docking sites for signaling proteins known as signal transducers and activators of transcription (STATs). JAKs catalyze STAT phosphorylation, which facilitates STAT dimerization and nuclear transport. The end result is the regulation of gene expression and transcription (Figure 1). The JAK/STAT pathway is a signal-transduction pathway resulting in lymphocyte cell cycle propagation and subsequent cell proliferation.

More specifically, JAK3, has a restrictive tissue distribution on hematopoietic cells and is associated only with the common gamma-chain. Indeed, genetic absence or mutation in either the common gamma-chain or JAK3 produces defects in lymphoid development leading to a severe combined immunodeficiency syndrome phenotype [8,10], characterized by the absence of T cells and natural killer cells and the maintenance of normal numbers of poorly functional B cells (T^–^B^+^NK^–^ SCID).

This property together with the nonredundancy of the JAK/STAT pathway would therefore be important features of an efficient novel immunosuppression based on JAK3 inhibition.

CP-690,550 (later named tazocitinib and finally tofacitinib) is an orally administered pyrrolo-pyrimidine JAK3 inhibitor [11,12] with a low inhibitory potency for JAK1 and JAK2 (thus avoiding bone marrow toxicity and especially anemia). Although CP-690,550 has been studied in various autoimmune disorders (psoriasis, Crohn’s disease, ulcerative colitis, rheumatoid arthritis, and so forth), we will focus on its indication in kidney transplantation firstly in animal models and then in human transplantation.

## Animal studies with CP-690,550

### Murine model

The *in vivo* efficacy of CP-690,550 was first studied in a murine model of heterotopic heart transplantation [11]. Animals treated with vehicle alone rejected their allografts within 12 days. In contrast, dosing with CP-690,550 resulted in a dose-dependent increase in survival of transplanted hearts. Although the drug was administered for only 28 days, the two higher dose groups had median survival times >60 days. These data indicated that CP-690,550 was able to suppress a robust *in vivo* allogeneic response.

### Nonhuman primates

Kidney transplantations were performed between mixed leukocyte reaction-mismatched, ABO blood group-matched cynomolgus monkeys [11,13]. Animals received either CP-690,550 (*n* = 18) at various levels of exposure or its vehicle (*n* = 3) and were killed at day 90 or earlier in cases of allograft rejection. The mean survival time in animals treated with CP-690,550 was significantly longer than that in control animals (53 ± 7 days vs. 7 ± 1 days, *P* <0.0003) and was positively correlated with drug exposure. Four treated animals were euthanized at 90 days with a normal renal function and low-grade rejection on final pathology. Occurrence of rejection was significantly delayed in treated animals (46 ± 7 days from transplantation vs. 7 ± 1 days in controls, *P* <0.0003). Persistent anemia, polyoma virus-like nephritis (*n* = 2), and urinary calcium carbonate accretions (*n* = 3) were seen in animals with high drug exposure. Natural killer cells and CD4^+^ and CD8^+^ T cells were significantly reduced in treated animals. Blood glucose, serum lipid levels, and arterial blood pressure were within normal range in treated animals, and no cancer was demonstrated. These data confirmed the immunosuppressive potential of CP-690,550.

Moreover, the immunomodulatory effects of CP-690,550 were studied *in vitro* and *in vivo* in nonhuman primates [14]. Pharmacodynamic assessments of lymphocyte activation, function, proliferation and phenotype were performed in three settings: *in vitro* in whole blood isolated from untransplanted cynomolgus monkeys, *in vivo* in blood from untransplanted cynomolgus monkeys dosed with CP-690,550 for 8 days, and *in vivo* in blood from transplanted cynomolgus monkeys immunosuppressed with CP-690,550. *In vitro* exposure to CP-690,550 resulted in a significant reduction of IL-2-enhanced IFNγ production by T cells, T-cell surface expression of CD25 and CD7l, and T-cell proliferative capacities. Similar results were observed in animals dosed with CP-690,550. In addition, transplanted animals displayed significant reduction of natural killer cell and T-cell numbers whereas CD8^+^ effector memory T-cell populations were unaffected.

Finally, addition of CP-690,550 to mycophenolate mofetil (MMF) significantly improved allograft survival [15]. In the same model, monkeys were treated orally twice a day with CP-690,550 together with MMF or MMF alone and were killed at day 90 or earlier due to allograft rejection. The mean survival time in animals treated with MMF alone was significantly extended in animals that concurrently received CP-690,550 (23 ± 1 days vs. 59.5 ± 9.8 days, *P* <0.02). Combination animals exposed to higher levels of CP-690,550 had a significantly better survival than animals that received less CP-690,550 (75.2 ± 8.7 days vs. 33.3 ± 12.6 days, *P* <0.02). Anemia and gastrointestinal intolerance was seen in combination therapy animals that otherwise did not show evidence of viral or bacterial infection besides signs consistent with subclinical pyelonephritis.

We concluded from these three studies that the potential of CP-690,550 (efficacy and safety profile) was in favor of beginning clinical trials in humans.

## Human studies with CP-690,550

### Phase 1 trial

In a dose-escalation study, the safety and tolerability effects on lymphocyte subsets, and pharmacokinetics of CP-690,550 when coadministered with MMF were firstly assessed in stable renal allograft recipients [16]. Twenty-eight patients were enrolled: six patients received CP-690,550 5 mg twice daily (BID), six patients received 15 mg BID, 10 patients received 30 mg BID, and six patients received placebo. The most frequent adverse events were infections and gastrointestinal symptoms (abdominal pain, diarrhea, dyspepsia, and vomiting). CP-690,550 15 mg BID and 30 mg BID were associated with a mean decrease in hemoglobin from baseline (11%), and a mean decrease in absolute natural killer cell counts (50%). CP-690,550 30 mg BID was also associated with a mean increase in absolute CD19^+^ B lymphocytes (130%). There were no changes in the number of neutrophils, total lymphocytes, platelets, or CD4^+^ or CD8^+^ T cells, clinical chemistry, vital signs, or electrocardiograms from the pretreatment baseline. Administration of CP-690,550 without a concomitant calcineurin inhibitor resulted in CP-690,550 exposures consistent with previous studies in nontransplant subjects.

As part of this study, the effect of CP-690,550 after 29 days of 30 mg BID treatment was investigated at the cellular level in eight kidney transplant recipients by studying *ex vivo* phosphorylation of STAT5, the key substrate of JAK3 [17]. As determined by quantitative fluorescent western blotting, IL-2-induced phosphorylated STAT5 in YT cells was reduced in the presence of serum collected on day 29 compared with pretreatment baseline. When evaluated by phosphospecific flow cytometry, CP-690,550 also reduced IL-2-induced phosphorylated STAT5 in CD3^+^, CD3^+^CD4^+^ and CD3^+^CD8^+^ patient blood subpopulations.

Finally, again as part of the initial study [16], blood samples were collected on day 1 (before first dose), day 15, day 29 (end of treatment), and day 57 [18]. Leukocyte counts remained stable, whereas a significant decrease in hemoglobin (8%) was documented. CP-690,550 treatment for 29 days resulted in statistically significant changes in the number of circulating CD19^+^ B cells (increased), CD3^+^CD16^+^CD56^+^ natural killer cells (decreased), and CD4^+^CD25^bright^ T cells (decreased). On day 15 after CP-690,550 treatment, the number of B cells increased (100%) whereas those of natural killer cells and CD4^+^CD25^bright^ T cells decreased (65% and 38%, respectively) from the pretreatment baseline. However, the regulatory capacities of the residual CD4^+^CD25^bright^ T cells remained unchanged. In addition, in the presence of CP-690,550, the IFNγ production capacity of peripheral blood mononuclear cells was reduced compared with the predose baseline.

### Phase 2a trial

Subsequently, a randomized pilot study compared CP-690,550 (15 mg BID and 30 mg BID, *n* = 20 in each group) with tacrolimus (*n* = 21) in *de novo* kidney allograft recipients [19]. Patients also received an IL-2 receptor antagonist, MMF and corticosteroids. CP-690,550 doses were reduced after 6 months. Due to a high incidence of BK virus nephropathy in CP30 patients, MMF was discontinued in this group. The 6-month biopsy-proven acute rejection rates were one out of 20, four out of 20 and one out of 21 for the 15 mg BID CP-690,550, 30 mg BID CP-690,550 and tacrolimus groups, respectively. BK virus nephropathy developed in four out of 20 patients in the 30 mg BID CP-690,550 group. The 6-month rates of cytomegalovirus disease were two out of 20, four out of 20 and none out of 21 for the 15 mg BID CP-690,550, 30 mg BID CP-690,550 and tacrolimus groups, respectively. The estimated glomerular filtration rate was >70 ml/minute at 6 and 12 months (all groups). In the CP-690,550 arms, there were modest lipid elevations and a trend toward more frequent anemia and neutropenia during the first 6 months. These data suggested that coadministration of 30 mg BID CP-690,550 with MMF was clearly associated with overimmunosuppression. At 15 mg BID, the efficacy/safety profile was comparable with that of the tacrolimus control group, except for a higher rate of viral infection (cycomegalovirus and BK virus).

In this study, the effect of CP-690,550 on IL-2-mediated JAK/STAT5 phosphorylation by CD4^+^CD25^bright^FoxP3^+^CD127^–/low^ regulatory T cells (Tregs) and CD4^+^CD25^–^ effector T cells (Teffs) was examined in kidney transplant patients [20]. Phosphospecific flow cytometry was used to study the effect of CP-690,550 on IL-2-induced intracellular STAT5 phosphorylation. IL-2-induced phosphorylation of STAT5 was significantly higher for CD4^+^CD25^bright^ Tregs than for CD4^+^CD25^–^ Teffs. In the presence of 100 ng/ml CP-690,550, a clinically relevant exposure, IL-2-induced phosphorylated STAT5 was partially inhibited in CD4^+^CD25^bright^ Tregs but was almost completely blocked in Teffs. The IC50 was two or three times higher for Tregs than for Teffs. In the presence of CP-690,550, Tregs exhibited additional suppressive activities on the alloactivated proliferation of Teffs. In addition, CD4^+^CD25^bright^ Tregs from kidney transplant patients receiving CP-690,550 vigorously suppressed the proliferation of Teffs.

CP-690,550 was therefore inhibiting Teff function but preserved the suppressive activity of CD4^+^CD25^bríght^ Tregs.

### Phase 2b trial

In a Phase 2b, randomized, multicenter, partially blinded, parallel group study [21], 331 low to moderate risk *de novo* kidney transplant patients were randomized to a more intensive or less intensive regimen of tofacitinib (CP-690,550) or cyclosporine. All patients also received basiliximab induction, MMF and corticosteroids. Co-primary noninferiority endpoints were incidence of the biopsy-proven acute rejection rate at month 6 or biopsy-proven rejection meeting serum creatinine criteria at month 6 (serum creatinine increase ≥0.3 mg/dl and ≥20% from pre-rejection baseline) and measured glomerular filtration rate at month 12.

Similar 6-month incidences of clinical biopsy-proven acute rejection (11%, 7% and 9%) were observed for the more intensive, less intensive and cyclosporine groups. Measured glomerular filtration rates were higher at month 12 for the more intensive and less intensive groups versus the cyclosporine group (65 ml/minute, 65 ml/ minute vs. 54 ml/minute). Fewer patients in the more intensive or less intensive groups developed chronic allograft nephropathy at month 12 compared with cyclosporine patients (25%, 24% vs. 48%). Serious infections (especially cytomegalovirus and BK virus) developed in 45%, 37% and 25% of more intensive patients, less intensive patients and cyclosporine patients, respectively.

Anemia, neutropenia and posttransplant lymphoproliferative disorder (four patients) occurred more frequently in more intensive and less intensive patients compared with cyclosporine patients. Tofacitinib was therefore equivalent to cyclosporine in preventing acute rejection, was associated with improved renal function and less chronic allograft histological injury, but developed serious side effects at the doses evaluated with an exposure-response relationship. This not unexpectedly high incidence of infectious complications is probably due to the broad JAK3-inhibited gamma-chain-dependent cytokines such as IL-2, IL-4, IL-7, IL-9, IL-15 and IL-21 [22]. This affects natural killer cells (IL-15) and the memory T-cell compartment. Patients receiving this compound therefore probably mount a less efficient primary antiviral response as well as a secondary one.

It would have been very interesting to try to keep the potent immunosuppressive effect together with the absence of nephrotoxicity either by avoiding high drug exposure, which seemed to correlate with infectious complications, or by modifying the companion drug or its dosage.

## Conclusion

Inhibiting JAK3 with tofacitinib was, and still is, clearly an attractive option in transplantation with a low incidence of rejection, no nephrotoxicity, and better histological preservation but a safety profile that was not ideal for low to moderate risk patients (more infections and cancers). Recently, following as yet unpublished data with a longer follow-up time, the development of tofacitinib had to be discontinued due mainly to a still higher incidence of infections in the treated group. As the number of high immunological risk patients is increasing steadily in most transplant center programs, it is unfortunate that this drug was not tested in such subgroups. Overall, this has been felt as a missed opportunity by the transplant community in need of new and better immunosuppressive drugs.

## Abbreviations

BID: twice daily; IFN: interferon; IL: interleukin; JAK: Janus kinase; MMF: mycophenolate mofetil; PCR: polymerase chain reaction; STAT: signal transducer and activator of transcription; Teff: effector T cell; Treg: regulatory T cell.

## Declaration

This article has been published as part of *Transplantation Research* Volume 2 Suppl 1, 2013: Proceedings of the 12th International Symposium on Transplantation. The full contents of the supplement are available at http://www.transplantationresearch.com/supplements/2/S1. The supplement is based on the 12th International Transplantation Symposium held on 16–17 April 2012, in Istanbul, Turkey. The symposium and publication based on these proceedings were supported by an unrestricted educational grant from Pfizer Inc., who had no influence on the editorial content. Editorial support was provided by Synergy, Richmond, UK and was funded by Pfizer Inc. Rapamune (sirolimus) is a product marketed by Pfizer Inc. The articles have been through the journal’s standard peer review process and the Supplement Editors declare that they have no competing interests.
